# Concurrent Assessment of Deformability and Adhesiveness of Sickle Red Blood Cells by Measuring Perfusion of an Adhesive Artificial Microvascular Network

**DOI:** 10.3389/fphys.2021.633080

**Published:** 2021-04-28

**Authors:** Madeleine Lu, Celeste K. Kanne, Riley C. Reddington, Dalia L. Lezzar, Vivien A. Sheehan, Sergey S. Shevkoplyas

**Affiliations:** ^1^Department of Biomedical Engineering, University of Houston, Houston, TX, United States; ^2^Division of Hematology/Oncology, Department of Pediatrics, Baylor College of Medicine, Houston, TX, United States

**Keywords:** deformability, adhesiveness, microvascular perfusion, capillary network, microfluidics, sickle cell disease, hemorheology, rheological biomarkers

## Abstract

Biomarker development is a key clinical research need in sickle cell disease (SCD). Hemorheological parameters are excellent candidates as abnormal red blood cell (RBC) rheology plays a critical role in SCD pathophysiology. Here we describe a microfluidic device capable of evaluating RBC deformability and adhesiveness concurrently, by measuring their effect on perfusion of an artificial microvascular network (AMVN) that combines microchannels small enough to require RBC deformation, and laminin (LN) coating on channel walls to model intravascular adhesion. Each AMVN device consists of three identical capillary networks, which can be coated with LN (adhesive) or left uncoated (non-adhesive) independently. The perfusion rate for sickle RBCs in the LN-coated networks (0.18 ± 0.02 nL/s) was significantly slower than in non-adhesive networks (0.20 ± 0.02 nL/s), and both were significantly slower than the perfusion rate for normal RBCs in the LN-coated networks (0.22 ± 0.01 nL/s). Importantly, there was no overlap between the ranges of perfusion rates obtained for sickle and normal RBC samples in the LN-coated networks. Interestingly, treatment with poloxamer 188 decreased the perfusion rate for sickle RBCs in LN-coated networks in a dose-dependent manner, contrary to previous studies with conventional assays, but in agreement with the latest clinical trial which showed no clinical benefit. Overall, these findings suggest the potential utility of the adhesive AMVN device for evaluating the effect of novel curative and palliative therapies on the hemorheological status of SCD patients during clinical trials and in post-market clinical practice.

## Introduction

Normal human red blood cells (RBCs) can squeeze through capillaries as small as 3 μm in diameter, and generally do not adhere to the endothelial cells that line blood vessels (Mohandas and Gallagher, 2008). Continual, unimpeded passage of RBCs through the microvasculature is crucial for maintaining appropriate levels of perfusion and tissue oxygenation (Lipowsky, 2005). In sickle cell disease (SCD), sickle RBCs exhibit both decreased deformability and increased adhesiveness due to a genetic defect that ultimately distorts the shape of the RBC and renders it more rigid. The change in shape has detrimental effects on microvascular perfusion (Mohandas and Evans, 1989; Relevy et al., 2008; Barabino et al., 2010; Hess, 2010; Da Costa et al., 2016), and results in a wide range of serious clinical complications, including pain crisis, end organ damage, and stroke (Matsui et al., 2001; Goel and Diamond, 2002; Byrnes and Wolberg, 2017). Additionally, while more deformable sickle RBCs are generally considered clinically favorable, as they are expected to flow more easily through the microvasculature, they are associated with greater levels of RBC adhesion and aggregation (Connes et al., 2013; Deng et al., 2019). Thus, the interplay between RBC deformability and adhesiveness with respect to their combined effect on microvascular blood flow is complex, and can make clinical interpretation of individual laboratory measurements challenging. Given the profoundly synergistic effect of both deformability and adhesion on blood flow, it is essential that we evaluate these two properties concurrently within a single device.

Currently, most devices for measuring RBC deformability and adhesion do so independently. While change in shape (elongation) of adherent RBCs under shear in adhesion assays provides some measure of deformability, such a measurement omits the contribution of all non-adherent RBCs and does not account for the wide range of deformations RBCs experience in the microcirculation (Relevy et al., 2008; Alapan et al., 2016b). The channels of microfluidic networks lined with endothelial cells invariably must have diameters larger than 15–20 μm to enable confluent culture; this trade-off omits the deformations RBCs experience in the smallest capillaries (Carden et al., 2017). Conversely, unlined microfluidic devices with narrow, capillary-size channels (some as small as 3–5 μm in diameter) designed to evaluate various aspects of RBC deformability do not assess adhesion (Glodek et al., 2010; Du et al., 2015; Picot et al., 2015; Burns et al., 2016; Islamzada et al., 2020; Man et al., 2020; Robidoux et al., 2020).

Here we present the development and initial validation of an artificial microvascular network (AMVN) that allows concurrent assessment of deformability and adhesiveness of RBCs isolated from whole blood and re-suspended at a physiologic hematocrit. The device measures the ability of RBCs to traverse a network of channels ranging from 5 μm “capillaries” to a 70 μm-wide “venule” to mimic the wide range of deformations RBCs experience *in vivo*. Additionally, the walls of the AMVN microchannels are coated with a relevant adhesion molecule to measure the effect of RBC adhesiveness on the network perfusion. We have previously developed a non-adhesive version of the AMVN (Shevkoplyas et al., 2006; Burns et al., 2012), which we used extensively to evaluate the effect of various parameters on microvascular network perfusion, including RBC deformability (Shevkoplyas et al., 2006; Burns et al., 2012; Sosa et al., 2014; Piety et al., 2021), hematocrit (Piety et al., 2017; Reinhart et al., 2017a), RBC aggregation (Reinhart et al., 2017b), RBC shape (Piety et al., 2016), and osmolality of the suspending medium (Reinhart et al., 2015b). In this study, we coated the adhesive AMVN with laminin (LN), a ligand that mediates the interaction between the basal cell adhesion molecule (BCAM)/Lutheran (Lu) on the surface of sickle RBCs and the sub-endothelium matrix. LN becomes exposed when the endothelium is damaged, and is therefore particularly relevant to a vasculopathy like SCD (Bartolucci et al., 2010; Laurance et al., 2011). We validated our new system by comparing the AMVN perfusion rate for samples of sickle (HbSS) and healthy (HbAA) RBCs, with and without LN coating. We then tested the utility of the system to detect drug response by measuring the effect of poloxamer 188 (P188), a tri-block copolymer known for its ability to repair cell membrane damage (Maskarinec et al., 2002; Moloughney and Weisleder, 2012). P188 was found to have a beneficial effect on rheological behavior of sickle RBCs in other *in vitro* assays (Gibbs and Hagemann, 2004; Sandor et al., 2016), and was investigated extensively as a potential therapy option for SCD (Orringer et al., 2001; Ballas et al., 2004), ultimately failing to reduce the frequency and duration of vaso-occlusive crises (VOC) in a Phase III clinical trial (EPIC, 2016).

## Materials and Methods

### Fabrication of the Microfluidic Device

**FIGURE 1:**
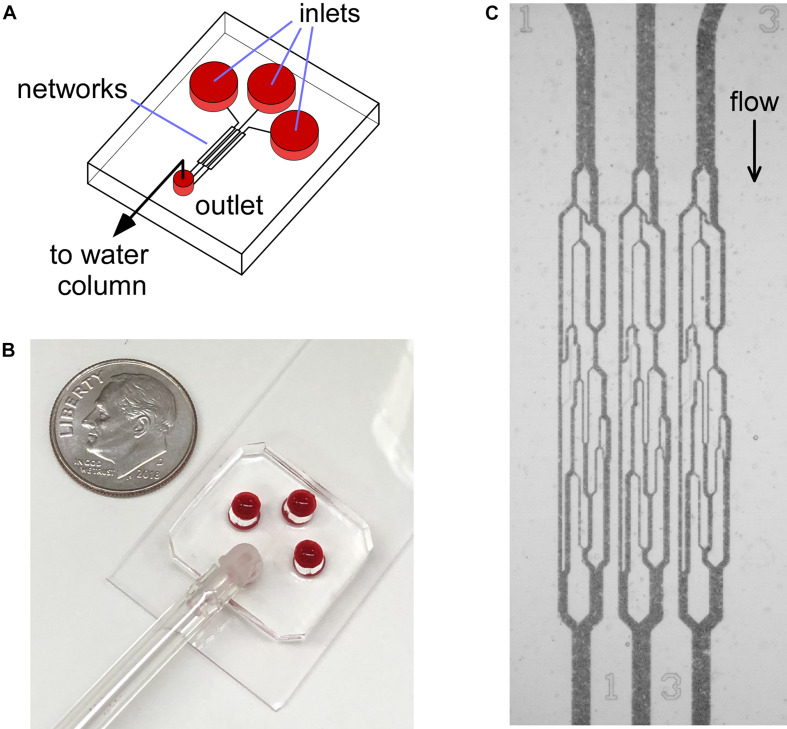
Illustration of the AMVN device setup. **(A)** Each device consists of three identical capillary networks, connecting three separate inlets to a common outlet. **(B)** Photograph of the AMVN device. Ten cent U.S. coin is shown for size reference. **(C)** A bright-field microscopy image showing the flow of blood in the capillary networks of an AMVN device. The device is illuminated through a blue filter to increase contrast (RBCs appear dark in blue light).

The design and fabrication of the non-adhesive, uncoated AMVN has been previously described in detail (Burns et al., 2012, 2016). Briefly, a master wafer patterned with the layout of the AMVN device was fabricated using photolithography, and polydimethylsiloxane (PDMS; Sylgard 184, Dow Corning Corp, Midland, MI) mixed at a 1:10 (curing agent to liquid PDMS) was poured onto the master wafer and allowed to cure (3 h at 65°C). Once cured, the AMVN replicas were peeled off the master wafers, plasma-oxidized (100 s, air plasma, PDC-3xG, Harrick Plasma, Ithaca, NY), and then bonded to a glass slide coated with PDMS. Each individual AMVN device consisted of three identical networks with three separate inlets, leading to a single, common outlet (Figure 1; Burns et al., 2012). Within 1 h of oxidation and bonding of the AMVN device, the “non-adhesive” networks of the device were filled with a 1% mPEG-silane (Laysan Bio, Inc., Arab, AL) solution in GASP buffer (1.3 mmol/L NaH_2_PO_4_, 9 mmol/L Na_2_HPO_4_, 140 mmol/L NaCl, 5.5 mmol/L glucose, 1% bovine serum albumin; osmolality 290 mmol/kg, pH 7.4). The “adhesive” networks of the device were treated similarly to a previously published method (Alapan et al., 2016a; Kim et al., 2017), by filling and incubating for 1 h at room temperature (RT) with a solution of heterobifunctional cross-linker Sulfo N-g-Maleimidobutyryloxy succinimide ester (Sulfo-GMBS, 1mM in saline; Thermo Fisher Scientific, Waltham, MA), and then rinsing with saline to remove unbound cross-linker. Next, the networks were filled with a solution of LN (sourced from Engel-Holm-Swarm murine sarcoma basement membrane; Sigma-Aldrich, St. Louis, MO) that was diluted with normal saline at 1:10 (LN to saline by volume) (Alapan et al., 2016a). Following 1 h incubation at RT, the “adhesive” networks were flushed with saline to remove unbound LN. Finally, all networks of the device were flushed with 1% mPEG-saline solution in GASP buffer and incubated overnight at 4°C, to minimize non-specific adhesion. Prior to running each experiment, the devices were flushed with normal saline for 1 min.

### Blood Samples

Whole blood was collected from SCD patients with the HbSS genotype (*n* = 7 female, *n* = 6 male; 1–20 years of age; HbS = 21.6–85.9%; HbF = 0.7–32.9%, HbA = 0–66.3%) during their regular clinic visits to the Texas Children’s Hospital (Houston, TX) into 3 mL vacutainer tubes (K_2_EDTA, BD), under a Baylor College of Medicine Institutional Review Board approved protocol. The %HbS, %HbF, %HbA, genotype, treatment type, gender, and age were obtained by review of patient’s medical records (see Supplementary Information for detailed patient information). Whole blood from normal individuals was collected from consenting volunteers into 4 mL vacutainer tubes (K_2_EDTA, BD), under a University of Houston Institutional Review Board approved protocol. Blood samples from both healthy volunteers and SCD patients were leukocyte- and platelet-reduced by removing the buffy coat and platelet-rich plasma after gravity sedimentation at RT, and the RBCs were then re-suspended in normal saline at either 25% or 40% hematocrit (HCT). For Poloxamer 188 (P188) experiments, RBCs were incubated with 5 or 10 mg/mL P188 (Kolliphor P188; CAS: 9003-11-6; Sigma-Aldrich, St. Louis, MO) at 25% HCT for 30 min at 37°C, following a previously published protocol (Sandor et al., 2016).

### Measurements of the AMVN Perfusion Rate

**FIGURE 2:**
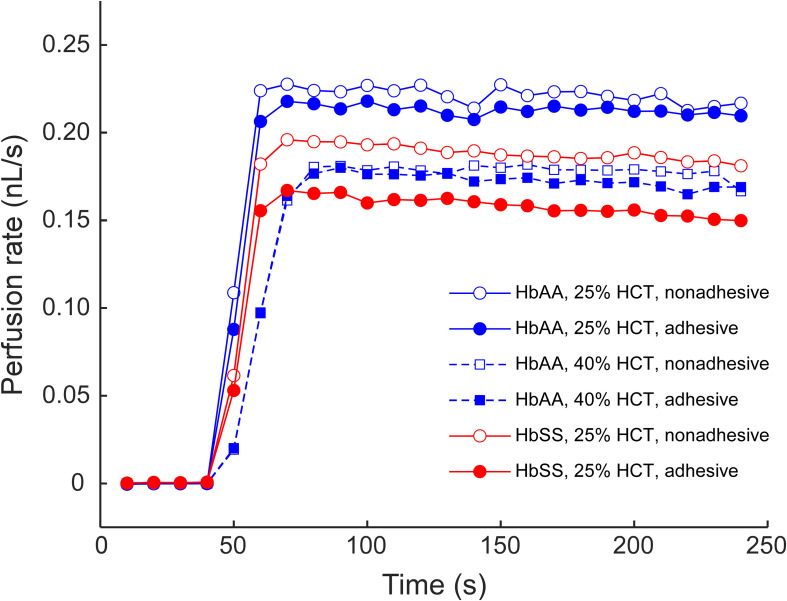
An example of the AMVN perfusion rate traces for samples of sickle (HbSS) and normal (HbAA) RBCs at 25 and 40% HCT, in either adhesive (LN) or non-adhesive (mPEG) networks. A constant HCT of approximately 25% was chosen for all subsequent experiments.

**FIGURE 3:**
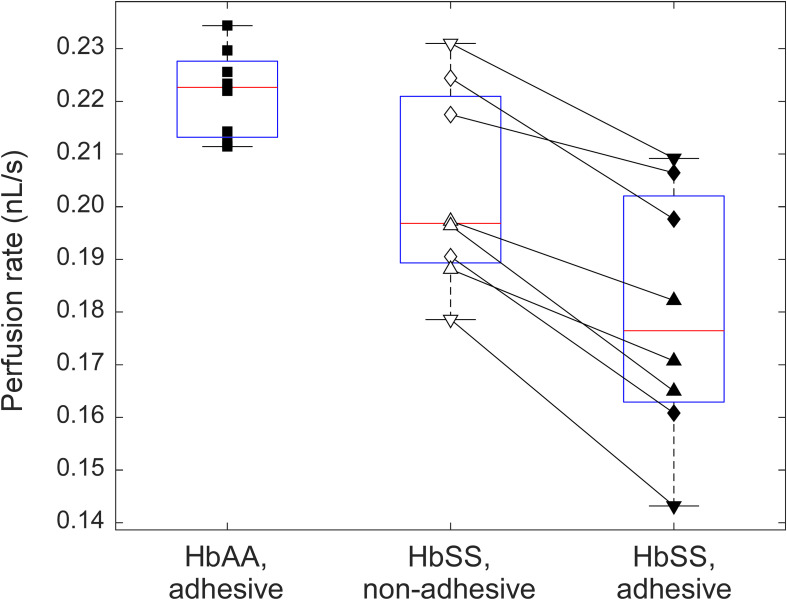
A box-plot comparison between the perfusion rates in adhesive (LN) and non-adhesive (mPEG) networks of the AMVN device for samples of normal (HbAA, *n* = 8) and sickle (HbSS, *n* = 8) RBCs suspended at 25% HCT. HbAA samples are indicated by squares. For samples from SCD patients (HbSS), triangles denote treatment with hydroxyurea, upside down triangles denote treatment with transfusion therapy, and rhombi denote treatment with both hydroxyurea and transfusion. All differences between the groups are statistically significant (*p* < 0.05).

**FIGURE 4:**
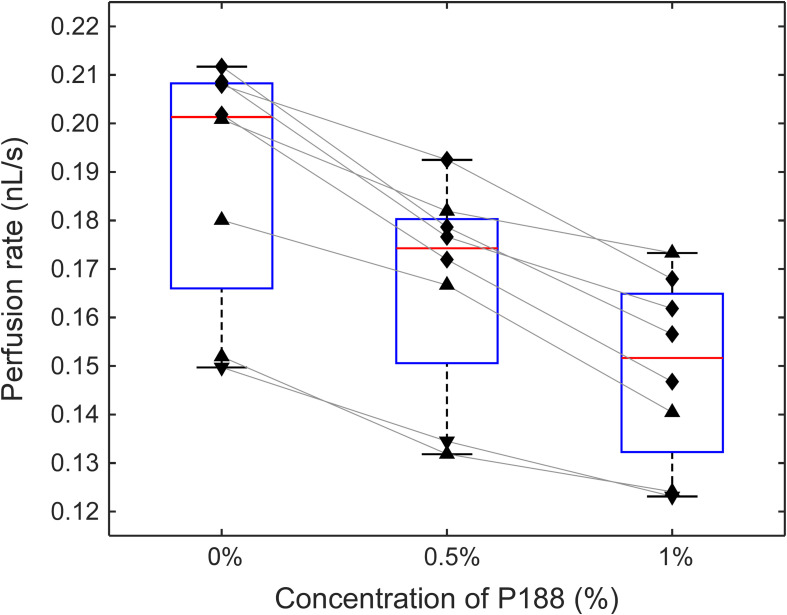
The effect of incubation of sickle RBCs with P188 at a concentration of 0 (control), 0.5, and 1% (w/v) on perfusion of the adhesive AMVN (*n* = 8 for each condition). Triangles denote treatment with hydroxyurea, upside down triangles denote treatment with transfusion therapy, and rhombi denote treatment with both hydroxyurea and transfusion. Differences between all groups were statistically significant (*p* < 0.5, paired *t*-test).

The experimental setup used to measure the AMVN perfusion rates has been previously described in detail (Shevkoplyas et al., 2006; Burns et al., 2012, 2016; Sosa et al., 2014). In a typical AMVN experiment, each network of the device was first filled with the RBC sample by applying a small pressure difference between the inlets and the outlet to drive the flow. The pressure difference was then brought to zero (confirmed by observing cessation of movement of RBCs in the channels), and the recording of the images for measuring the network perfusion was initiated. Finally, the pressure difference was set to −20 cm H_2_O, and remained constant for the duration of the experiment (3 min). The flow of RBCs in the AMVN was observed using an inverted microscope (iX83, Olympus America, Inc., Center Valley, PA) equipped with a high-speed camera (Flea3, Point Gray Research, Inc., Richmond, Canada) to acquire images of the post-AMVN (venular) channels (10-image bursts at 100 fps every 10 s). Images were analyzed using a custom image analysis algorithm implemented in MATLAB (The Math Works Inc., Natick, MA) to determine the average RBC velocity in each venular channel; the network perfusion rate was then calculated by multiplying the average RBC velocity by the area of the venular channel cross section. During an experiment, the perfusion rate increased rapidly soon after the driving pressure was applied to the device, reaching a plateau once the flow was fully established (<1 min, for an example see Figure 2). The overall perfusion rate was calculated by averaging over the plateau region (e.g., Figures 3, 4). For additional details on the AMVN device design, fabrication and perfusion rate measurements please see Burns et al. (2012).

### Viscosity Measurements

The change in viscosity of the suspending medium due to the addition of P188 was evaluated by dissolving P188 at 0, 0.5, and 1% w/v in DI water, and measuring the viscosity of each solution on a cone/plate viscometer (DV3T, Brookfield Engineering Laboratories, Inc., Middleboro, MA). All measurements were performed in triplicate at 25°C and a constant shear rate of 225 (1/s).

### Statistical Analysis

The statistical significance of the differences between the perfusion rates of adhesive and non-adhesive AMVN for the same subject was evaluated using paired *t*-test. The differences in AMVN perfusion rates between groups of subjects were compared using two-sample *t*-test. Differences with *p*-value of less than 0.05 were considered significant. All data analysis was performed using functions of the Statistics and Machine Learning Toolbox in MATLAB. All values are shown as mean ± standard deviation.

## Results

### Design of the Adhesive Artificial Microvascular Network Device

Figure 1 illustrates the design and operation of the adhesive AMVN device. Because the device consisted of three identical, individually addressable microchannel networks, we were able to treat each of the three networks independently, changing the number of adhesive and non-adhesive network units within the same AMVN device to match the needs of a particular experiment. To make a network “non-adhesive,” we treated the channels with mPEG-silane following the same protocol we implemented in our earlier studies for minimizing non-specific adhesion (Burns et al., 2012; Forouzan et al., 2012). To make a network “adhesive,” we coated the walls of its microchannels with LN by adapting a method previously published in the literature (Alapan et al., 2016a). Figure 2 shows examples of the perfusion rate traces measured for a sample of sickle RBCs (at native HCT of ∼25%) and normal RBCs from a healthy volunteer (with HCT adjusted to either 25 or 40%), in both adhesive and non-adhesive networks. The AMVN perfusion rate for normal RBCs showed a strong dependence on sample HCT, as expected. For each sample (either sickle or normal), the perfusion rate in the adhesive network was consistently lower than in the non-adhesive network, although this difference was substantially larger for sickle RBCs compared to normal RBCs. Interestingly, the perfusion rate for sickle RBC sample (25% HCT) in the adhesive network was about the same as for normal RBC sample (40% HCT) in either adhesive or non-adhesive network, notwithstanding the rather significant difference in HCT in favor of the sickle RBC sample. Finally, the perfusion rate for sickle RBCs in non-adhesive network was substantially faster than that in adhesive network, but still much slower than that of normal RBCs with the same 25% HCT, regardless of adhesion (Figure 2).

### Additive Effect of Deformability and Adhesiveness on Microvascular Network Perfusion for Sickle RBCs

In order to validate our system’s ability to detect the differences in deformability and adhesiveness of normal and sickle RBCs, we fabricated AMVN devices with one non-adhesive and two adhesive networks to compare side-by-side the flow behavior of normal RBCs and sickle RBCs in the presence and absence of LN-mediated adhesion, under the same flow conditions. Figure 3 compares the perfusion rate of normal RBCs (*n* = 8, HCT = 25.4 ± 0.5%) in one of the adhesive networks of the device, with that of sickle RBCs (*n* = 8, HCT = 25.4 ± 0.7%) in the non-adhesive and in the other adhesive network of the same device (the values shown in Figure 3 were obtained by averaging over the plateau region of the perfusion rate traces measured in each experiment, see for example Figure 2, as described previously; Burns et al., 2012). On average, the perfusion rate for sickle RBCs in the adhesive network (0.18 ± 0.02 nL/s) was significantly (paired *t*-test; *p* < 0.05) lower than that in the non-adhesive network (0.20 ± 0.02 nL/s), and both were significantly (two-sample *t*-test; *p* < 0.05) lower than the perfusion rate for normal RBCs (0.22 ± 0.01 nL/s). For each sickle RBC sample, regardless of the subject’s therapy, the perfusion rate in the adhesive network was lower than in non-adhesive network by 11.8 ± 4.9%, on average (range: 5.1–19.7%). The range of perfusion rates measured for samples from different SCD patients in either adhesive (range: 0.14–0.21 nL/s, 13% coefficient of variation/CV) or non-adhesive (range: 0.18–0.23 nL/s, 9% CV) network was substantially wider than for samples from healthy subjects (range: 0.21–0.23 nL/s, 4% CV). Importantly, there was no overlap of the perfusion rate in the adhesive AMVN between normal and sickle RBCs. Each sample was run on three different devices, and had an average CV of less than 5%, indicating excellent reproducibility of perfusion rate values from the same sample across three different devices (data not shown).

### Effect of P188 on the Ability of Sickle RBCs to Perfuse an Adhesive Microvascular Network

Figure 4 shows the effect of increasing concentrations of P188 on the ability of sickle RBCs to perfuse an adhesive AMVN. In these experiments the AMVN devices were fabricated with all three networks coated with LN, which allowed for a side-by-side comparison of HbSS RBC samples (25% HCT) treated with two different concentrations of P188 and an untreated control. We found an 11.7 ± 3.5% (range: 7.4–15.6%) decrease in the perfusion rate for sickle RBCs treated with 0.5% P188 compared to the untreated control (paired *t*-test, *p* = 0.00011). There was a further decrease of 10.4 ± 4.1% (range: 4.8–15.7%) in perfusion rate for sickle RBCs treated with 1% P188 (paired *t*-test, *p* = 0.00037), leading to an overall decrease of approximately 21.0 ± 4.5% (range: 13.7–27.3%) relative to the untreated control (paired *t*-test, *p* = 0.00003). The viscosity of the suspending medium, measured with a cone/plate viscometer, increased from 0.97 ± 0.01 cP to 1.01 ± 0.01 cP (5% increase) for 0.5% P188, and further to 1.16 ± 0.05 cP (20% increase) for 1.0% P188 (*p* < 0.05 for all differences).

## Discussion

Endpoints in current clinical trials evaluating new therapies for SCD typically include a combination of conventional hematologic parameters (e.g., total Hb, HbF level) and observed clinical complications (e.g., frequency of VOC). However, not every novel SCD therapy produces a change in conventional laboratory values (e.g., crizanlizumab, L-glutamine) (Kucukal et al., 2020; Lu et al., 2020). Additionally, monitoring for clinical complications (e.g., counting VOC over a period of time) is a slow and subjective process. Finally, none of these common endpoints assess RBC functional properties, and hence cannot determine if the therapy has reduced the rheological abnormalities associated with SCD. Incorporating hemorheological biomarkers (RBC deformability and adhesiveness) into the clinical trial protocols alongside traditional clinical endpoints has the potential to provide a much more informative, mechanistic, and less subjective assessment of the effectiveness of novel SCD therapies (Kucukal et al., 2020; Lu et al., 2020).

At present, these two RBC properties are typically assessed *in vitro* using separate devices, which entirely overlooks the complex interplay between the effect of deformability and adhesiveness on microvascular blood flow observed *in vivo*. Deformability is typically measured using classic ektacytometry (Connes et al., 2014, 2018; Parrow et al., 2017) and filtration assays (Smith et al., 1987), as well as various microfluidic devices (Burns et al., 2012; Picot et al., 2015; Guzniczak et al., 2018; Islamzada et al., 2020; Robidoux et al., 2020). Adhesiveness is evaluated by quantifying RBC adhesion to cultured endothelium or specific adhesion molecules under static (Smith et al., 1987) and flow conditions (Montes et al., 2002; Relevy et al., 2008; Alapan et al., 2016b). These classical adhesion assays can provide a measure of deformability, but only for the adherent RBCs and under a very limited set of flow conditions (Relevy et al., 2008; Alapan et al., 2016b). Even the cutting-edge microfluidic systems lined with cultured endothelium (to better mimic the complexity of RBC adhesion to the vessel wall) are limited to channels larger than 15-20 μm, omitting the deformations RBCs experience in the smallest capillaries (Mannino et al., 2015; Carden et al., 2017). To our knowledge, the adhesive AMVN is the first device that can measure *in vitro* how the basic functional properties of sickle RBCs may affect perfusion of capillary networks at native HCT and under physiological flow conditions. By measuring the perfusion rate of the same sickle RBC sample in both an LN-coated and a non-adhesive network under the normoxic conditions, we were able to comprehensively assess the synergistic effect of both deformability and adhesiveness on perfusion rate. We observed that the perfusion rate of all sickle RBC samples in our study significantly decreased in the presence of LN, independent of deformability (as literally the same sample was ran in a LN-coated and a non-adhesive network of the same device under the same conditions simultaneously). Our results indicate the importance of examining deformability within the context of adhesion rather than as an independent metric.

We observed a much wider range of the AMVN perfusion rate values for RBC samples from SCD patients than for samples from HbAA individuals, which agrees well with previous studies (Smith et al., 1987; Udani et al., 1998; Maciaszek et al., 2014; Alapan et al., 2016a, b). Sickle RBC samples in our study were taken from patients on a variety of therapeutic interventions (hydroxyurea, transfusion, or both) and it is well-known that patient responses to treatment vary greatly (Lu et al., 2020). For example, patients on transfusion therapy would have a highly heterogeneous mixture of their own (sickle) RBCs and of normal RBCs donated by multiple donors and preserved in hypothermic storage for up to 6 weeks prior to transfusion. Clearly, the exact composition of the blood sample would vary greatly from one patient to another, as well as for the same patient between treatments. Additionally, the degree to which the AMVN perfusion rate is reduced by the storage-induced deterioration of rheological properties of normal RBCs differs significantly among donors (Burns et al., 2012; Reinhart et al., 2015a; Piety et al., 2021). It is likely therefore that both the differences in individual patient’s response to a therapy and transfusion frequency, as well as the heterogeneity of the RBC population (i.e., presence of both host sickle RBCs and normal stored RBCs) in the sample contributed to greater variability of perfusion rates for SCD patients we observed in this study. Future studies with a larger cohort of patients on various treatment options are needed to further elucidate how current therapies effect microvascular perfusion rate as a function of both deformability and adhesiveness.

Notwithstanding the relatively high variability observed for samples from SCD patients, there was no significant overlap between perfusion rates for sickle and normal RBCs in the adhesive AMVN. That is, our device could distinguish between samples from HbAA individuals with presumably normal blood rheology, and samples from SCD patients all of whom were receiving treatment aimed at correcting hemorheological abnormalities caused by SCD. Interestingly, the adhesive AMVN was able to make this distinction even under normoxic conditions, and even for samples of sickle RBCs with HbS content as low as 22%. These findings suggest that the rheological status of SCD patients may be still compromised even if their therapy is highly effective at significantly reducing the level of HbS. Additional research will be needed to determine if the ability of the adhesive AMVN (and its future improved designs) to distinguish between normal and sickle RBCs will hold for a larger number of patients and normal controls.

Another potential explanation for the perfusion rate variability is that it may be difficult to completely deplete the RBC suspensions of leukocytes and platelets, particularly in the case of SCD patient samples that often come in relatively small volumes. The presence of leukocytes and platelets may affect the measured perfusion rate (Forouzan et al., 2012), and therefore care should be taken to remove them as much as possible and count the residual leukocytes and platelets in the samples to make sure the results are not dependent on the concentration of contaminating cells. Similarly, HCT must be carefully equalized across all compared samples because of the strong dependence of AMVN perfusion rate on HCT of the sample (Piety et al., 2017; Reinhart et al., 2017a). We found no significant correlation between sample leukocyte and platelet concentrations and perfusion rate (data not shown), which suggests that sample preparation was not a significant factor in this study.

We chose to evaluate P188 using our new adhesive AMVN device because the effect of this molecule on RBC properties has been investigated over the years using a wide range of *in vitro* assays designed to measure either RBC deformability or adhesiveness separately. Early studies showed that incubation with P188 can improve deformability of sickle RBCs in the classical Nuclepore filtration and micropipette aspiration assays (Smith et al., 1987). However, more recent studies using ektacytometry (Connes et al., 2014) and a microfluidic filtration assay (Picot et al., 2015) showed no improvement in deformability of sickle RBCs after the P188 treatment (Sandor et al., 2016). In contrast, while early studies showed that P188 has no effect on deformability of normal RBCs in the filtration/micropipette aspiration assays (Smith et al., 1987), more recent studies showed that P188 had a positive effect on deformability (measured via ektacytometry) of normal RBCs stored in DEHP-free/ethylene vinyl acetate bags (Cancelas et al., 2017), but produced significant stiffening of normal RBCs (measured using a microfluidic device) after a short term incubation (Guzniczak et al., 2018). Reports on the effect of P188 on adhesiveness of sickle RBCs have been more consistent, showing a significantly reduced adhesion of sickle (but not of normal) RBCs to cultured endothelial cells (Smith et al., 1987; Sandor et al., 2016). P188 was also found to significantly reduce blood viscosity and aggregation of sickle RBCs in some assays, supporting the notion of an overall positive effect on the rheological behavior of sickle RBCs (Gibbs and Hagemann, 2004; Sandor et al., 2016). None of the previous studies of P188’s effects on RBC rheology investigated its effects on microvascular perfusion in either non-adhesive or adhesive networks, nor did they evaluated the changes in deformability and adhesiveness concurrently. Regardless, because of these positive findings *in vitro*, P188 has been tested extensively as a potential therapy option for SCD (Adams-Graves et al., 1997; Orringer et al., 2001; Ballas et al., 2004).

In this study, we found a significant concentration-dependent decline of the perfusion rate in the adhesive AMVN for sickle RBCs treated with P188. The perfusion rate in our system depends on RBC properties (e.g., deformability and adhesiveness) as well as on a number of other relevant hemorheological parameters, including viscosity of the suspending medium. Therefore, if there was any improvement in RBC deformability or adhesiveness due to P188, it was insufficient to overcome the increased viscosity of the suspending medium caused by P188, resulting in an overall reduction of the perfusion rate. Our results agree well with early studies that reported elevated plasma and whole blood viscosity for samples treated with similar concentrations of P188 (Lechmann and Reinhart, 1998). The lack of a favorable effect of P188 on microvascular perfusion in our simulated capillary network is also in agreement with the latest Phase III clinical trial for P188 in SCD (EPIC, NCT01737814), which failed to show any significant benefit with the treatment group reporting a slightly (although not statistically significantly) higher mean duration of VOC (82 h) than placebo group (78 h) (EPIC, 2016). These results highlight the importance of further developing and validating comprehensive functional assays for assessing the rheological properties of RBCs *in vitro*, particularly in the context of novel potentially curative therapies for SCD (Lu et al., 2020). Reliance on assays designed to probe only a single aspect of RBC rheological behavior may lead to an over-interpretation of narrow *in vitro* data, missing the bigger picture that might indicate a potential lack of efficacy and/or adverse outcomes. The use of comprehensive assays—such as the adhesive AMVN device—may help improve the initial screening of promising drug candidates to hopefully avoid Phase III clinical trial failures, like those seen for P188 (Ataga et al., 2011; EPIC, 2016).

AMVN is an artificial microvascular network comprising a relatively small number of rectangular microchannels made of PDMS, and therefore it has several well-known limitations which make it difficult to completely mimic the complex microvascular environment *in vivo* (Shevkoplyas et al., 2003, 2006; Sosa et al., 2014). The simplicity of network architecture, rectangular shape of channel cross-section and the mismatch of material properties may limit the ability of the AMVN to simulate some aspects of microvascular blood flow dynamics (Shevkoplyas et al., 2005; Forouzan et al., 2011; Yang et al., 2011; Fenech et al., 2019). *In vivo*, sickle RBCs adhere to LN (chosen in this study to simulate adhesion to subendothelial matrix exposed due to endothelial damage) as well as to several other adhesion molecules expressed on the surface of endothelial cells (Kaul et al., 2009; Alapan et al., 2016a), which are missing in our current proof-of-concept system. Finally, our sample preparation procedure requires depletion of leukocytes and platelets, and a careful adjustment of sample HCT, which resulted in a substantial reduction of plasma proteins in the suspending medium. However, both leukocyte and platelets, as well as plasma proteins, are known to affect the microvascular blood flow and to mediate the interactions of circulating blood cells with endothelium. The sensitivity of our assay to sample preparation quality is an important limitation. Ultimately, integrating the required sample preparation into the device, or accounting for the effect of sample composition on perfusion rate in the design of the experiments could help mitigate this drawback.

In conclusion, our adhesive AMVN device allows evaluating both RBC deformability and adhesiveness concurrently, through their effect on perfusion of a network of capillary-size microchannels coated with relevant adhesion molecules. The AMVN is fabricated from PDMS, a well-studied biocompatible polymer, which can be either rendered non-adhesive or functionalized to mimic the adhesive properties of inflamed endothelium or exposed sub-endothelial matrix. This flexibility allows exploring a much wider range of interactions of sickle RBCs with the vessel wall, far beyond the BCAM/Lu-mediated adhesion to LN we investigated in this proof-of-concept study. Future research employing the adhesive AMVN device for screening new drug candidates for their effect on RBC adhesiveness and deformability, as well as in longitudinal studies for evaluating the effectiveness of novel therapies, could have a significant positive impact on health and wellbeing of SCD patients worldwide.

## Data Availability Statement

The raw data supporting the conclusions of this article will be made available by the authors, without undue reservation.

## Ethics Statement

The studies involving human participants were reviewed and approved by the Baylor College of Medicine Institutional Review Board and University of Houston Institutional Review Board. Written informed consent to participate in this study was provided by the participants’ legal guardian/next of kin.

## Author Contributions

ML, VS, and SS designed the study and wrote the manuscript. ML, RR, and DL fabricated the microfluidic devices and performed the experiments. CK facilitated collection of patient samples and performed initial sample preparation. SS and VS supervised the project. All authors critically reviewed and approved the manuscript.

## Conflict of Interest

SS was an inventor of the artificial microvascular network technology described in this article, and has received compensation as a consultant for Hemanext (formerly New Health Sciences, Inc.) which is commercializing this technology. The remaining authors declare that the research was conducted in the absence of any commercial or financial relationships that could be construed as a potential conflict of interest.

## References

[B1] Adams-Graves P. Kedar A. Koshy M. Steinberg M. Veith R. Ward D. (1997). RheothRx (poloxamer 188) injection for the acute painful episode of sickle cell disease: a pilot study. *Blood* 90 2041–2046. 10.1182/blood.v90.5.2041 9292541

[B2] Alapan Y. Kim C. Adhikari A. Gray K. E. Gurkan-Cavusoglu E. Little J. A. (2016a). Sickle cell disease biochip: a functional red blood cell adhesion assay for monitoring sickle cell disease. *Transl. Res.* 173:e8. 10.1016/j.trsl.2016.03.008 PMC4959913 27063958

[B3] Alapan Y. Matsuyama Y. Little J. A. Gurkan U. A. (2016b). Dynamic deformability of sickle red blood cells in microphysiological flow. *Technology* 4 71–79. 10.1142/s2339547816400045 27437432 PMC4947547

[B4] Ataga K. I. Reid M. Ballas S. K. Yasin Z. Bigelow C. James L. S. (2011). Improvements in haemolysis and indicators of erythrocyte survival do not correlate with acute vaso-occlusive crises in patients with sickle cell disease: a phase III randomized, placebo-controlled, double-blind study of the Gardos channel blocker senicapoc (ICA-17043). *Br. J. Haematol.* 153 92–104. 10.1111/j.1365-2141.2010.08520.x 21323872

[B5] Ballas S. K. Files B. Luchtman-Jones L. Benjamin L. Swerdlow P. Hilliard L. (2004). Safety of purified poloxamer 188 in sickle cell disease: phase I study of a non-ionic surfactant in the management of acute chest syndrome. *Hemoglobin.* 28 85–102. 10.1081/hem-120035919 15182051

[B6] Barabino G. A. Platt M. O. Kaul D. K. (2010). Sickle cell biomechanics. *Annu. Rev. Biomed. Eng.* 12 345–367. 20455701 10.1146/annurev-bioeng-070909-105339

[B7] Bartolucci P. Chaar V. Picot J. Bachir D. Habibi A. Fauroux C. (2010). Decreased sickle red blood cell adhesion to laminin by hydroxyurea is associated with inhibition of Lu/BCAM protein phosphorylation. *Blood* 116 2152–2159. 10.1182/blood-2009-12-257444 20566895

[B8] Burns J. M. Yang X. Forouzan O. Sosa J. M. Shevkoplyas S. S. (2012). Artificial microvascular network: a new tool for measuring rheologic properties of stored red blood cells. *Transfusion* 52 1010–1023. 10.1111/j.1537-2995.2011.03418.x 22043858

[B9] Burns J. M. Yoshida T. Dumont L. J. Yang X. Piety N. Z. Shevkoplyas S. S. (2016). Deterioration of red blood cell mechanical properties is reduced in anaerobic storage. *Blood Transfus.* 14 80–88. 26674833 10.2450/2015.0241-15 PMC4731343

[B10] Byrnes J. R. Wolberg A. S. (2017). Red blood cells in thrombosis. *Blood* 130 1795–1799. 10.1182/blood-2017-03-745349 28811305 PMC5649548

[B11] Cancelas J. A. Rugg N. Nestheide S. Hill S. E. Emanuele R. M. McKenzie D. S. (2017). The purified vepoloxamer prevents haemolysis in 42-day stored, DEHP/PVC-free red blood cell units. *Blood Transfus.* 15 165–171. 28263175 10.2450/2017.0351-16 PMC5336339

[B12] Carden M. A. Fay M. E. Lu X. Mannino R. G. Sakurai Y. Ciciliano J. C. (2017). Extracellular fluid tonicity impacts sickle red blood cell deformability and adhesion. *Blood* 130 2654–2663. 10.1182/blood-2017-04-780635 28978568 PMC5731085

[B13] Connes P. Lamarre Y. Hardy-Dessources M. D. Lemonne N. Waltz X. Mougenel D. (2013). Decreased hematocrit-to-viscosity ratio and increased lactate dehydrogenase level in patients with sickle cell anemia and recurrent leg ulcers. *PLoS One.* 8:e79680. 10.1371/journal.pone.0079680 24223994 PMC3817120

[B14] Connes P. Lamarre Y. Waltz X. Ballas S. K. Lemonne N. Etienne-Julan M. (2014). Haemolysis and abnormal haemorheology in sickle cell anaemia. *Br. J. Haematol.* 165 564–572. 10.1111/bjh.12786 24611951

[B15] Connes P. Renoux C. Romana M. Abkarian M. Joly P. Martin C. (2018). Blood rheological abnormalities in sickle cell anemia. *Clin. Hemorheol. Microcirc.* 68 165–172. 29614630 10.3233/CH-189005

[B16] Da Costa L. Suner L. Galimand J. Bonnel A. Pascreau T. Couque N. (2016). Diagnostic tool for red blood cell membrane disorders: Assessment of a new generation ektacytometer. *Blood Cells Mol. Dis.* 56 9–22. 10.1016/j.bcmd.2015.09.001 26603718 PMC4811191

[B17] Deng Y. Papageorgiou D. P. Chang H. Y. Abidi S. Z. Li X. Dao M. (2019). Quantifying Shear-Induced Deformation and Detachment of Individual Adherent Sickle Red Blood Cells. *Biophys. J.* 116 360–371. 10.1016/j.bpj.2018.12.008 30612714 PMC6350013

[B18] Du E. Diez-Silva M. Kato G. J. Dao M. Suresh S. (2015). Kinetics of sickle cell biorheology and implications for painful vasoocclusive crisis. *Proc. Natl. Acad. Sci. U S A.* 112 1422–1427. 10.1073/pnas.1424111112 25605910 PMC4321273

[B19] EPIC (2016). *Evaluation of Purified Poloxamer 188 in Vaso-Occlusive Crisis of Sickle Cell Disease (EPIC) (EPIC) [Internet].*. Available from: https://clinicaltrials.gov/ct2/show/NCT01737814 (accessed date July 22, 2020)

[B20] Fenech M. Girod V. Claveria V. Meance S. Abkarian M. Charlot B. (2019). Microfluidic blood vasculature replicas using backside lithography. *Lab. Chip.* 19 2096–2106. 10.1039/c9lc00254e 31086935

[B21] Forouzan O. Burns J. M. Robichaux J. L. Murfee W. L. Shevkoplyas S. S. (2011). Passive recruitment of circulating leukocytes into capillary sprouts from existing capillaries in a microfluidic system. *Lab. Chip.* 11 1924–1932. 10.1039/c0lc00547a 21503282

[B22] Forouzan O. Yang X. Sosa J. M. Burns J. M. Shevkoplyas S. S. (2012). Spontaneous oscillations of capillary blood flow in artificial microvascular networks. *Microvasc. Res.* 84 123–132. 10.1016/j.mvr.2012.06.006 22732344

[B23] Gibbs W. J. Hagemann T. M. (2004). Purified poloxamer 188 for sickle cell vaso-occlusive crisis. *Ann. Pharmacother.* 38 320–324. 10.1345/aph.1d223 14742772

[B24] Glodek A. M. Mirchev R. Golan D. E. Khoory J. A. Burns J. M. Shevkoplyas S. S. (2010). Ligation of complement receptor 1 increases erythrocyte membrane deformability. *Blood* 116 6063–6071. 10.1182/blood-2010-04-273904 20861458 PMC3031392

[B25] Goel M. S. Diamond S. L. (2002). Adhesion of normal erythrocytes at depressed venous shear rates to activated neutrophils, activated platelets, and fibrin polymerized from plasma. *Blood* 100 3797–3803. 10.1182/blood-2002-03-0712 12393714

[B26] Guzniczak E. Jimenez M. Irwin M. Otto O. Willoughby N. Bridle H. (2018). Impact of poloxamer 188 (Pluronic F-68) additive on cell mechanical properties, quantification by real-time deformability cytometry. *Biomicrofluidics* 12:044118. 10.1063/1.5040316 PMC6404947 30867863

[B27] Hess J. R. (2010). Red cell changes during storage. *Transfus Apher. Sci.* 43 51–59. 10.1016/j.transci.2010.05.009 20558107

[B28] Islamzada E. Matthews K. Guo Q. Santoso A. T. Duffy S. P. Scott M. D. (2020). Deformability based sorting of stored red blood cells reveals donor-dependent aging curves. *Lab Chip.* 20 226–235. 10.1039/c9lc01058k 31796943

[B29] Kaul D. K. Finnegan E. Barabino G. A. (2009). Sickle red cell-endothelium interactions. *Microcirculation* 16 97–111. 10.1080/10739680802279394 18720225 PMC3059190

[B30] Kim M. Alapan Y. Adhikari A. Little J. A. Gurkan U. A. (2017). Hypoxia-enhanced adhesion of red blood cells in microscale flow. *Microcirculation* 24:5. 10.1111/micc.12374 PMC5679205 28387057

[B31] Kucukal E. Man Y. Hill A. Liu S. Bode A. An R. (2020). Whole blood viscosity and red blood cell adhesion: Potential biomarkers for targeted and curative therapies in sickle cell disease. *Am. J. Hematol.* 2020:25933. 10.1002/ajh.25933 PMC7689825 32656816

[B32] Laurance S. Lansiaux P. Pellay F. X. Hauchecorne M. Benecke A. Elion J. (2011). Differential modulation of adhesion molecule expression by hydroxycarbamide in human endothelial cells from the micro- and macrocirculation: potential implications in sickle cell disease vasoocclusive events. *Haematologica.* 96 534–542. 10.3324/haematol.2010.026740 21228039 PMC3069230

[B33] Lechmann T. Reinhart W. H. (1998). The non-ionic surfactant Poloxamer 188 (RheothRx) increases plasma and whole blood viscosity. *Clin. Hemorheol. Microcirc.* 18 31–36. 9653583

[B34] Lipowsky H. H. (2005). Microvascular rheology and hemodynamics. *Microcirculation* 12 5–15. 10.1080/10739680590894966 15804970

[B35] Lu M. Rab M. A. Shevkoplyas S. S. Sheehan V. A. (2020). Blood rheology biomarkers in sickle cell disease. *Exp. Biol. Med.* 245 155–165. 10.1177/1535370219900494 31948290 PMC7016416

[B36] Maciaszek J. L. Partola K. Zhang J. Andemariam B. Lykotrafitis G. (2014). Single-cell force spectroscopy as a technique to quantify human red blood cell adhesion to subendothelial laminin. *J. Biomech.* 47 3855–3861. 10.1016/j.jbiomech.2014.10.016 25458578

[B37] Man Y. Kucukal E. An R. Watson Q. D. Bosch J. Zimmerman P. A. (2020). Microfluidic assessment of red blood cell mediated microvascular occlusion. *Lab. Chip.* 20 2086–2099. 10.1039/d0lc00112k 32427268 PMC7473457

[B38] Mannino R. G. Myers D. R. Ahn B. Wang Y. Margo R. Gole H. (2015). Do-it-yourself in vitro vasculature that recapitulates in vivo geometries for investigating endothelial-blood cell interactions. *Sci. Rep.* 5:12401. 10.1038/srep12401 PMC4894411 26202603

[B39] Maskarinec S. A. Hannig J. Lee R. C. Lee K. Y. (2002). Direct observation of poloxamer 188 insertion into lipid monolayers. *Biophys. J.* 82 1453–1459. 10.1016/s0006-3495(02)75499-4 11867460 PMC1301946

[B40] Matsui N. M. Borsig L. Rosen S. D. Yaghmai M. Varki A. Embury S. H. P. - (2001). selectin mediates the adhesion of sickle erythrocytes to the endothelium. *Blood* 98 1955–1962. 10.1182/blood.v98.6.1955 11535535

[B41] Mohandas N. Evans E. (1989). Rheological and adherence properties of sickle cells. Potential contribution to hematologic manifestations of the disease. *Ann. N Y Acad. Sci.* 565 327–337. 10.1111/j.1749-6632.1989.tb24180.x 2672968

[B42] Mohandas N. Gallagher P. G. (2008). Red cell membrane: past, present, and future. *Blood* 112 3939–3948. 10.1182/blood-2008-07-161166 18988878 PMC2582001

[B43] Moloughney J. G. Weisleder N. (2012). Poloxamer 188 (p188) as a membrane resealing reagent in biomedical applications. *Recent Pat. Biotechnol.* 6 200–211. 10.2174/1872208311206030200 23092436 PMC3756676

[B44] Montes R. A. Eckman J. R. Hsu L. L. Wick T. M. (2002). Sickle erythrocyte adherence to endothelium at low shear: role of shear stress in propagation of vaso-occlusion. *Am. J. Hematol.* 70 216–227. 10.1002/ajh.10145 12111767

[B45] Orringer E. P. Casella J. F. Ataga K. I. Koshy M. Adams-Graves P. Luchtman-Jones L. (2001). Purified poloxamer 188 for treatment of acute vaso-occlusive crisis of sickle cell disease: A randomized controlled trial. *JAMA.* 286 2099–2106. 10.1001/jama.286.17.2099 11694150

[B46] Parrow N. L. Tu H. Nichols J. Violet P. C. Pittman C. A. Fitzhugh C. (2017). Measurements of red cell deformability and hydration reflect HbF and HbA2 in blood from patients with sickle cell anemia. *Blood Cells Mol. Dis.* 65 41–50. 10.1016/j.bcmd.2017.04.005 28472705

[B47] Picot J. Ndour P. A. Lefevre S. D. El Nemer W. Tawfik H. Galimand J. (2015). A biomimetic microfluidic chip to study the circulation and mechanical retention of red blood cells in the spleen. *Am. J. Hematol.* 90 339–345. 10.1002/ajh.23941 25641515

[B48] Piety N. Z. Reinhart W. H. Pourreau P. H. Abidi R. Shevkoplyas S. S. (2016). Shape matters: the effect of red blood cell shape on perfusion of an artificial microvascular network. *Transfusion* 56 844–851. 10.1111/trf.13449 26711854 PMC4833558

[B49] Piety N. Z. Reinhart W. H. Stutz J. Shevkoplyas S. S. (2017). Optimal hematocrit in an artificial microvascular network. *Transfusion* 57 2257–2266. 10.1111/trf.14213 28681482 PMC5583001

[B50] Piety N. Z. Stutz J. Yilmaz N. Xia H. Yoshida T. Shevkoplyas S. S. (2021). Microfluidic capillary networks are more sensitive than ektacytometry to the decline of red blood cell deformability induced by storage. *Sci. Rep.* 11:604. 10.1038/s41598-020-79710-3 PMC7804960 33436749

[B51] Reinhart W. H. Piety N. Z. Shevkoplyas S. S. (2017a). Influence of feeding hematocrit and perfusion pressure on hematocrit reduction (Fahraeus effect) in an artificial microvascular network. *Microcirculation* 24:8. 10.1111/micc.12396 PMC5673536 28801994

[B52] Reinhart W. H. Piety N. Z. Shevkoplyas S. S. (2017b). Influence of red blood cell aggregation on perfusion of an artificial microvascular network. *Microcirculation* 24:5. 10.1111/micc.12317 PMC5357595 27647727

[B53] Reinhart W. H. Piety N. Z. Deuel J. W. Makhro A. Schulzki T. Bogdanov N. (2015a). Washing stored red blood cells in an albumin solution improves their morphologic and hemorheologic properties. *Transfusion* 55 1872–1881. 10.1111/trf.13052 25752902 PMC4536109

[B54] Reinhart W. H. Piety N. Z. Goede J. S. Shevkoplyas S. S. (2015b). Effect of osmolality on erythrocyte rheology and perfusion of an artificial microvascular network. *Microvasc. Res.* 98 102–107. 10.1016/j.mvr.2015.01.010 25660474 PMC4361376

[B55] Relevy H. Koshkaryev A. Manny N. Yedgar S. Barshtein G. (2008). Blood banking-induced alteration of red blood cell flow properties. *Transfusion* 48 136–146. 17900281 10.1111/j.1537-2995.2007.01491.x

[B56] Robidoux J. Laforce-Lavoie A. Charette S. J. Shevkoplyas S. S. Yoshida T. Lewin A. (2020). Development of a flow standard to enable highly reproducible measurements of deformability of stored red blood cells in a microfluidic device. *Transfusion* 60 1032–1041. 10.1111/trf.15770 32237236 PMC9701565

[B57] Sandor B. Marin M. Lapoumeroulie C. Rabai M. Lefevre S. D. Lemonne N. (2016). Effects of Poloxamer 188 on red blood cell membrane properties in sickle cell anaemia. *Br. J. Haematol.* 173 145–149. 10.1111/bjh.13937 26846309

[B58] Shevkoplyas S. S. Gifford S. C. Yoshida T. Bitensky M. W. (2003). Prototype of an in vitro model of the microcirculation. *Microvasc. Res.* 65 132–136. 10.1016/s0026-2862(02)00034-1 12686171

[B59] Shevkoplyas S. S. Yoshida T. Gifford S. C. Bitensky M. W. (2006). Direct measurement of the impact of impaired erythrocyte deformability on microvascular network perfusion in a microfluidic device. *Lab. Chip.* 6 914–920. 10.1039/b601554a 16804596

[B60] Shevkoplyas S. S. Yoshida T. Munn L. L. Bitensky M. W. (2005). Biomimetic autoseparation of leukocytes from whole blood in a microfluidic device. *Anal. Chem.* 77 933–937. 10.1021/ac049037i 15679363 PMC3022340

[B61] Smith C. M. II Hebbel R. P. Tukey D. P. Clawson C. C. White J. G. Vercellotti G. M. (1987). Pluronic F-68 reduces the endothelial adherence and improves the rheology of liganded sickle erythrocytes. *Blood* 69 1631–1636. 3580571

[B62] Sosa J. M. Nielsen N. D. Vignes S. M. Chen T. G. Shevkoplyas S. S. (2014). The relationship between red blood cell deformability metrics and perfusion of an artificial microvascular network. *Clin. Hemorheol. Microcirc.* 57 275–289. 10.3233/ch-131719 23603326 PMC3766416

[B63] Udani M. Zen Q. Cottman M. Leonard N. Jefferson S. Daymont C. (1998). Basal cell adhesion molecule/lutheran protein. The receptor critical for sickle cell adhesion to laminin. *J. Clin. Invest.* 101 2550–2558. 10.1172/jci1204 9616226 PMC508844

[B64] Yang X. Forouzan O. Burns J. M. Shevkoplyas S. S. (2011). Traffic of leukocytes in microfluidic channels with rectangular and rounded cross-sections. *Lab. Chip.* 11 3231–3240. 10.1039/c1lc20293f 21847500

