# Supplementary material for: Concurrent Assessment of Deformability and Adhesiveness of Sickle Red Blood Cells by Measuring Perfusion of an Adhesive Artificial Microvascular Network
Source: Front Physiol. 2021 Apr 28;12:633080. doi: 10.3389/fphys.2021.633080 (PMC8113687; doi:10.3389/fphys.2021.633080)
Supplement: Supplementary file 1 [file Table_1.DOCX]

**Supplementary Information**

**Concurrent assessment of deformability and adhesiveness of sickle red blood cells by measuring perfusion of an adhesive artificial microvascular network**

Madeleine Lu^1^, Celeste K. Kanne^2,3^, Riley C. Reddington^1^, Dalia L. Lezzar^1^,
Vivien A. Sheehan^2,3,*^, Sergey S. Shevkoplyas^1,*^

**Table S1:** Hemoglobin profile, treatment, age, and gender of the SCD patient subjects.

|  | **HbS(%)** | **HbF(%)** | **HbA(%)** | **Treatment** | **Age** | **Gender** | **Figure** |
| --- | --- | --- | --- | --- | --- | --- | --- |
| 1 | 28.40 | 7.50 | 61.10 | HU+TF | 3 | M | 3 |
| 2 | 48.70 | 1.90 | 46.60 | TF | 15 | F | 3 |
| 3 | 85.80 | 10.90 | 0.00 | HU | 15 | M | 3 |
| 4 | 21.6 | 8.8 | 66.3 | HU+TF | 6 | M | 3 |
| 5 | 85.9 | 11.3 | 0 | HU | 3 | F | 3 |
| 6 | 40.8 | 0.7 | 54.2 | TF | 11 | F | 3 |
| 7 | 83.2 | 14 | 0 | HU | 7 | F | 3 & 4 |
| 8 | 59.9 | 19.1 | 17.9 | HU+TF | 3 | M | 3 & 4 |
| 9 | 46.6 | 8.60 | 41.60 | HU+TF | 3 | M | 4 |
| 10 | 64.90 | 32.90 | 0.00 | HU | 5 | F | 4 |
| 11 | 77.30 | 20.40 | 0.00 | HU | 2 | F | 4 |
| 12 | 55.20 | 1.20 | 41.10 | TF | 20 | M | 4 |
| 13 | 57.30 | 17.90 | 22.20 | HU+TF | 7 | F | 4 |
| 14 | 25.30 | 12.30 | 60.00 | HU+TF | 1 | M | 4 |
